# Validation of a home safety questionnaire used in a series of case-control studies

**DOI:** 10.1136/injuryprev-2013-041006

**Published:** 2014-03-03

**Authors:** Michael Watson, Penny Benford, Carol Coupland, Rose Clacy, Paul Hindmarch, Gosia Majsak-Newman, Toity Deave, Denise Kendrick

**Affiliations:** 1 Faculty of Medicine and Health Sciences, University of Nottingham, Nottingham, UK; 2 Institute of Health & Society, Newcastle University, Newcastle upon Tyne, UK; 3 Norfolk and Norwich University Hospital NHS Foundation Trust, Norwich, UK; 4 Faculty of Health and Applied Sciences, University of the West of England, Bristol, UK

**Keywords:** Methodology

## Abstract

**Objective:**

To measure the validity of safety behaviours, safety equipment use and hazards reported on a questionnaire by parents/carers with children aged under 5 years participating in a series of home safety case-control studies.

**Methods:**

The questionnaire measured safety behaviours, safety equipment use and hazards being used as exposures in five case-control studies. Responses to questions were compared with observations made during a home visit. The researchers making observations were blind to questionnaire responses.

**Results:**

In total, 162 families participated in the study. Overall agreement between reported and observed values of the safety practices ranged from 48.5% to 97.3%. Only 3 safety practices (stair gate at the top of stairs, stair gate at the bottom of stairs, stairs are carpeted) had substantial agreement based on the κ statistic (k=0.65, 0.72, 0.74, respectively). Sensitivity was high (≥70%) for 19 of the 30 safety practices, and specificity was high (≥70%) for 20 of the 30 practices. Overall for 24 safety practices, a higher proportion of respondents over-reported than under-reported safe practice (negative predictive value>positive predictive value). For six safety practices, a higher proportion of respondents under-reported than over-reported safe practice (negative predictive value<positive predictive value).

**Conclusions:**

This study found that the validity of self-reports varied with safety practice. Questions with a high specificity will be useful for practitioners for identifying households who may benefit from home safety interventions and will be useful for researchers as measures of exposures or outcomes.

## Introduction

Self-administered questionnaires have been extensively used in injury prevention research and evaluation.^1^ ^2^ They can be used as the sole research instrument (eg, in descriptive epidemiological studies) or as just one tool within research, such as RCTs or case-control studies.^3^ Questionnaires have also been used by practitioners, for example, in order to identify those who may benefit from home safety interventions.^4^ However they are used, it is crucial that care is taken with planning, and that they are rigorously designed.^5–9^

A key issue in survey research is validity, and concerns have been raised that self-reported safety practices might overestimate safe behaviour.^10^ ^11^ Measures with few false positives will be useful for practitioners for identifying those who may benefit from home safety interventions and for researchers, as high levels of specificity have been found to minimise bias in estimates of treatment effects in trials.^12^ More recently, different types of survey methods have been tested including face-to-face interviews, telephone interviews and researcher administered questionnaires; with considerable variation in their findings.^13–20^ It is important to note that these studies also varied in terms of topics covered; number of questions; timing of the observations in relation to the self-report and settings for the self-report.

There have been few empirical studies that validated self-administered questionnaires in child home injury prevention. One small study (n=64) investigating a range of home safety topics, found a fairly high degree of consistency between self-reported and observed practices.^21^ Another small study (n=30) of poison prevention practices found sensitivity, specificity and predictive value of self-reported possession, safe storage of, and exposure to substances varied between substances.^22^ It is of note that both studies took place within one city in England, and findings need confirmation from larger studies and with different populations.

Accordingly, our study aimed to validate a questionnaire comparing self-reported practices with home observations. The questionnaire was completed by parents or carers with children aged under five participating in five large multicentre case-control studies of home safety.^23^ The critical function of the questionnaire in the research was to measure safety behaviours, safety equipment use and hazards, which were used as exposures in the case-control studies investigating modifiable risk factors for falls, poisoning and scalds.

## Methods

The case-control studies recruited cases aged 0–4 years attending emergency departments (ED), minor injury units, or admitted to hospital in National Health Service (NHS) trusts with stairway falls, falls on the same level, falls from furniture, poisonings or suspected poisonings, or thermal injuries where the injury occurred in the home or garden.^23^ Cases were recruited from NHS trusts in and around four study centres in Nottingham, Bristol, Norwich and Newcastle-upon-Tyne, with recruitment commencing in June 2010. Controls were matched to the cases by age and sex, and recruited from general practices in the same study areas.

Data on exposures (safety behaviours, safety equipment use and hazards) and on potential confounding factors (sociodemographic and economic information, child health, development and behaviour, maternal mental health, parenting daily hassles) were collected by parent-completed questionnaires.^23^ Three age-specific questionnaires were developed (age 0–12 months, 13–36 months and 37–59 months). Case questionnaires were piloted on 11 parents/carers of children attending EDs at participating NHS Trusts, and control questionnaires were piloted on 29 parents/carers attending local children's centres. Questionnaires were checked for comprehension and ease of completion by a lay research advisor. For the case-control studies, most questionnaires were posted to participants and returned by post, but among cases a small number were completed in the ED with a researcher. All parents participating in the case-control studies were asked whether they would be interested in taking part in further research.

The answers to 78 of the questionnaire items which could be ascertained by observation were assessed during home visits to a subset of case-control study participants who expressed interest in taking part in further research. Potential participants were contacted by phone and visits were organised as soon as possible following receipt of the completed questionnaire. Respondents receiving home visits were given a £5 shopping voucher to thank them for their time.

A checklist was designed for completion during the home visit including exposures relating to the kitchen, stairs, use of infant equipment, and safe storage of medicines and household products. Home visits were conducted by one or two members from four research centre teams, blind to participants’ questionnaire responses. Participants were not told that they were taking part in a validation study: the home visit was explained in terms of finding out more about home safety generally.

Participants were asked to guide the researcher(s) on a tour of their home as required by the checklist, during which observations were made of the relevant safety behaviours, safety equipment use and hazards. This included, where appropriate, measurements of stair steepness and width of the biggest gap between banister rails. As well as conducting observations of current exposures, researchers asked about changes pertaining to exposures which had been made within the previous 3 months.

Data were entered into an access database, verified by double data entry and analysed using StataSE11. For the statistical analysis the answers to some questions were combined to categorise certain practices as ‘assumed to be safe’ or ‘potentially unsafe’ giving 31 binary exposures in total (shown in tables 2–4); for example, all medicines stored safely (yes/no), all household products stored safely (yes/no), all medicines and household products stored safely (yes/no). Storage was categorised as ‘safe’ if either there were no medicines/household products in the house, or if they were all stored at adult eye level or above, and/or in locked cupboards, cabinets, drawers or fridges. There were two additional safety variables which were width of the largest gap between banister rails and stair steepness (categorised as ‘too steep’ or ‘not too steep’ using questionnaire responses, and expressed as a ratio of stair height divided by stair depth using measurements from the home visit).

### Sample size

The sample size was calculated based on an estimated sensitivity of 80% (the number of participants reporting a specific exposure divided by the number observed to have the exposure). Assuming a minimum of 20% of participants had the exposure, and to estimate the sensitivity with a 95% CI of ±20%, then 16 exposed participants would be needed and so 80 home visits were required. This would enable a specificity of 80% to be estimated to within ±9.8%, assuming 80% of participants did not have the exposure. Since sensitivity and specificity may vary between cases and controls, 80 cases and 80 controls were required across the four study centres.

### Statistical analysis

**Figure 1:**
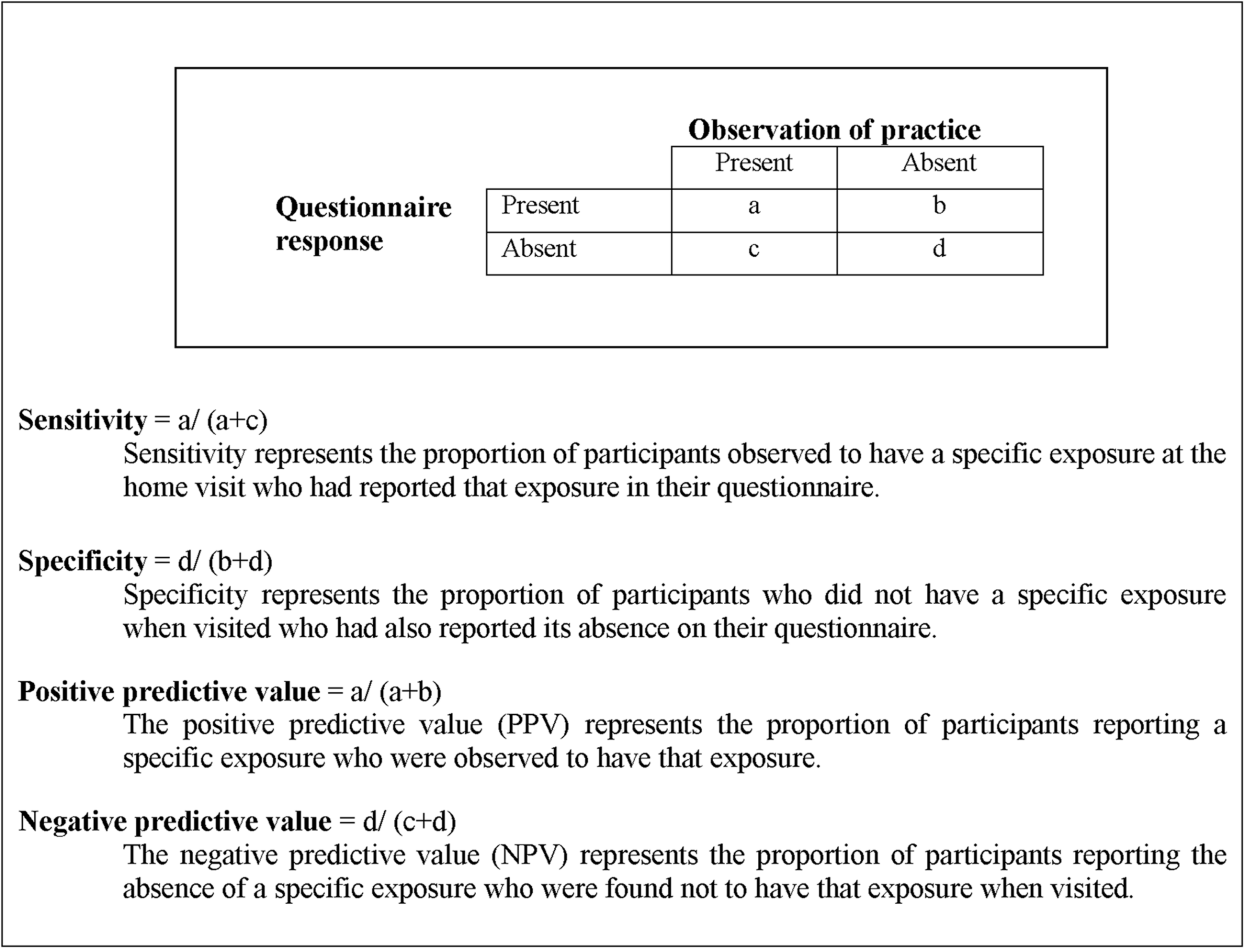
Key terms.

We calculated a number of different statistics to compare reported values with values observed at the home visit. For binary variables, overall percentage agreement and κ coefficients with 95% exact CIs were calculated. κ Values less than zero were considered to indicate poor agreement, 0–0.20 slight agreement, 0.21–0.40 fair agreement, 0.41–0.60 moderate agreement, 0.61–0.80 substantial agreement and 0.81–1.00 to reflect almost perfect agreement.^24^ Sensitivity, specificity, positive and negative predictive values (with 95% exact CIs) were calculated assuming observed values were the ‘true’ values (see figure 1).

For the analysis of the widest gap between stair banister railings, a median difference between observed and reported values was calculated, with an IQR and a Wilcoxon signed-rank test was performed. Stair steepness was calculated (defined as the height : depth ratio) based on measurements at the home visit and mean values compared between those reporting their stairs as too steep and those who did not with an unpaired t test.

The primary analysis compared responses on the questionnaires with the home observations. However, respondents may have made changes to their safety practices after completing the questionnaire and before the home visit. To assess whether differences between reported and observed practices may have arisen due to this, we asked about any changes made in the last 3 months during the home visit, and created a modified value for each exposure to reflect self-reported exposure at the time of the visit. If for any exposure the percentage of people reporting a change in the previous 3 months was more than 20% for any cell within the table comparing reported and observed values, the numbers were adjusted to accommodate an assumed change in reported values from yes to no and vice versa, and PPVs and NPVs were recalculated.

## Results

A total of 162 participants (81 controls and 81 cases) received home visits. The period of time between receipt of questionnaire and the visit being carried out varied between 1 and 92 days, the median being 29 days. Table 1 shows the characteristics of families participating in the home observations and case and control study participants who did not have a home observation. For most characteristics, there appears to be no significant difference between those participating in the home observations and the cases and controls that did not participate in home observations. However, respondents with children aged under 1 year were less likely and those with children aged 13–36 months were more likely to take part in the home observations. Respondents with male children, those in single parent families, and those in households with more adults out of work were also more likely to participate in the home observations.

**Table 1:** Characteristics of families observed at home and case-control study participants not observed at home

Characteristic	Home observation (n=162)	Cases/controls not observed at home (n=3289)	p Value
Age of child			<0.001
0–12 months	20 (12.4)	954 (29.0)	
13–36 months	107 (66.1)	1550 (47.1)	
37–62 months	35 (21.6)	785 (23.9)	
Sex of child: male	103 (63.6)	1823 (55.4)	0.041
Ethnic origin: white	150 (92.6)	2958 (91.5) [57]	0.63
Number of children aged <5 years in family		[46]	0.46
0	0	29 (0.9)	
1	91 (56.2)	1938 (59.8)	
2	64 (39.5)	1146 (35.3)	
≥3	7 (4.3)	130 (4.0)	
Case or control is first child	67 (43.5) [8]	1366 (45.0) [254]	0.72
Sex of respondent: female	156 (96.3)	3043 (92.5)	0.071
Maternal age ≤19 at birth of first child*	20 (13.0) [2]	295 (9.8) [22]	0.19
Single adult household	29 (17.9)	353 (11.0) [76]	0.007
Adults out of work		[61]	<0.001
0	77 (47.5)	1783 (55.2)	
1	44 (27.2)	1007 (31.2)	
≥2	41 (25.3)	438 (13.6)	
Receipt of state benefits	62 (38.8) [2]	1198 (37.4) [86]	0.73
Overcrowding (>1 person per room)	11 (7.1) [8]	228 (7.3) [175]	0.93
Non-owner occupier	64 (39.5)	1090 (33.7) [58]	0.13
Household has no car	26 (16.1)	378 (11.7) [50]	0.093
Median index of multiple deprivation score (IQR)	17.1 (9.5, 34.4)	15.2 (9.3, 27.3) [28]	0.068

Values are number (%) unless stated otherwise.

[ ] missing values.

*Only applicable if completed by mother.

In table 2, κ coefficients ranged from 0.2 (slight agreement) to 0.74 (substantial agreement) for safety practices relating to falls. However, there was substantial agreement for only three practices (has a stair gate at the top of stairs, has stair gate at the bottom of stairs, and, stairs are carpeted), whereas for nine practices the agreement was moderate or lower. Sensitivity was high (≥70%) for 8 of the 12 practices and specificity was high (≥70%) for 10 of the 12 practices.

**Table 2:** Sensitivity, specificity, predictive values, κ value and percentage agreement between the questionnaire responses and observations related to falls

Practice	Sensitivity (95% CI)	Specificity (95% CI)	PPV (95% CI)	NPV (95% CI)	κ Value (95% CI)	% Agreement
Has stair gate at top of stairs* [3]	90.4 (81.9 to 95.7)	73.8 (61.5 to 84.0)	81.5 (72.1 to 88.9)	85.7† (73.8 to 93.6)	0.65 (0.53 to 0.78)	83.1 (76.1 to 88.8)
Has stair gate at bottom of stairs* [6]	91.5 (81.3 to 97.2)	82.6 (72.9 to 89.9)	78.3 (66.7 to 87.3)	93.4† (85.3 to 97.8)	0.72 (0.61 to 0.83)	86.2 (79.5 to 91.4)
Has other safety gates in the house* [0]	42.1 (29.1 to 55.9)	95.7 (89.5 to 98.8)	85.7‡ (67.3 to 96.0)	73.2 (64.4 to 80.8)	0.42 (0.28 to 0.56)	75.5 (67.8 to 82.1)
Stairs are carpeted*[1]	98.6 (95.0 to 99.8)	75.0 (34.9 to 96.8)	98.6‡ (95.0 to 99.8)	75.0 (34.9 to 96.8)	0.74 (0.49 to 0.98)	97.3 (93.3 to 99.7)
Presence of landing part-way up stairs* [2]	73.3 (61.9 to 82.9)	85.1 (75.0 to 92.3)	83.3‡ (72.1 to 91.4)	75.9 (65.3 to 84.6)	0.58 (0.46 to 0.71)	79.2 (71.8 to 85.4)
Presence of banisters on all stairs*[8]	91.0 (82.4 to 96.3)	36.9 (25.3 to 49.8)	63.4 (53.8 to 72.3)	77.4† (58.9 to 90.4)	0.29 (0.15 to 0.43)	66.4 (58.1 to 74.1)
Presence of handrails on all stairs* [6]	87.7 (76.3 to 94.9)	51.1 (40.2 to 61.9)	53.8 (43.1 to 64.2)	86.5† (74.2 to 94.4)	0.35 (0.22 to 0.48)	65.5 (57.2 to 73.2)
Use of corner covers on any furniture [0]	70.6 (44.0 to 89.7)	85.5 (78.7 to 90.8)	36.4 (20.4 to 54.9)	96.1† (91.2 to 98.7)	0.40 (0.21 to 0.58)	84.0 (77.4 to 89.2)
Use of baby walker§ [1]	57.1 (28.9 to 82.3)	76.3 (67.4 to 83.8)	22.9 (10.4 to 40.1)	93.5† (86.5 to 97.6)	0.20 (0.03 to 0.38)	74.2 (65.7 to 81.5)
Use of stationary play centre§ [2]	60.0 (32.3 to 83.7)	82.1 (73.8 to 88.7)	31.0 (15.3 to 50.8)	93.9† (87.1 to 97.7)	0.30 (0.10 to 0.50)	79.5 (71.5 to 86.2)
Use of play pen§ [2]	80.0 (28.4 to 99.5)	95.9 (90.7 to 98.7)	44.4 (13.7 to 78.8)	99.2† (95.4 to 100)	0.55 (0.23 to 0.87)	95.3 (90.0 to 98.2
Use of travel cot instead of a playpen§ [1]	50.0 (18.7 to 81.3)	93.2 (87.1 to 97.0)	38.5 (13.9 to 68.4)	95.7† (90.1 to 98.6)	0.38 (0.11 to 0.65)	89.8 (83.3 to 94.5)

[ ] missing values.

*Questions only asked of those with stairs (n=151).

†A higher proportion of respondents over-reported than under-reported safe practice (NPV exceeds PPV).

‡A higher proportion of respondents under-reported than over-reported safe practice (PPV exceeds NPV).

§These practices were only asked for children aged ≤36 months (n=129).

There was no statistically significant difference (p=0.08) in measured stair steepness (stair height:depth ratio) between those reporting their stairs were too steep (n=23, mean=0.87, SD=0.21) and those who did not (n=121, mean=0.82, SD=0.09). Observed banister gaps were significantly larger than reported gaps (n=55, p=0.002). The median reported gap was 3.0 inches (IQR=2.0–4.0 inches) compared with a median observed gap of 3.8 inches (IQR=3.5–4.3 inches).

Table 3 shows the κ coefficients ranged from −0.03 to 0.54 for safety practices relating to poisonings. Only two practices had moderate agreement (κ values of 0.41–0.60) which were medicines kept in a fridge (κ=0.54), and all household products stored at adult eye level or above (κ=0.48). Sensitivity and specificity was high (≥70%) for 8 of the 15 practices.

**Table 3:** Sensitivity, specificity, predictive values, κ value and percentage agreement between the questionnaire responses and observations related to poisonings

Practice	Sensitivity (95% CI)	Specificity (95% CI)	PPV (95% CI)	NPV (95% CI)	κ Value (95% CI)	% Agreement (95% CI)
All medicines stored safely* [23]	83.8 (72.9 to 91.6)	31.0 (20.5 to 43.1)	53.8 (43.8 to 63.5)	66.7† (48.2 to 82.0)	0.15 (0.01 to 0.28)	56.8 (48.2 to 65.2)
All household products stored safely* [18]	75.9 (56.5 to 89.7)	60.9 (51.3 to 69.8)	32.8 (21.8 to 45.4)	90.9† (82.2 to 96.3)	0.25 (0.11 to 0.38)	63.9 (55.5 to 71.7)
All medicines and household products stored safely * [22]	68.8 (41.3 to 89.0)	68.5 (59.6 to 76.6)	22.0 (11.5 to 36.0)	94.4† (87.5 to 98.2)	0.19 (0.05 to 0.34)	68.6 (60.2 to 76.1)
All medicines stored in locked cupboard, cabinet, drawer or fridge [5]	0 (0 to 70.8)	87.7 (81.4 to 92.4)	0 (0 to 17.6)	97.8† (93.8 to 99.5)	−0.03 (−0.07 to 0.00)	86.0 (79.6 to 91.0)
All household products stored in locked cupboard, cabinet, drawer or fridge [8]	54.5 (23.4 to 83.3)	79.0 (71.4 to 85.4)	16.7 (6.4 to 32.8)	95.8† (90.4 to 98.6)	0.16 (0.00 to 0.33)	77.3 (69.8 to 83.6)
All medicines and household products stored in locked cupboard, cabinet, drawer or fridge [3]	*Unable to calculate due to frequencies of 0 in some cells*					92.5 (87.2 to 96.0)
All medicines stored at adult eye level or above [27]	78.1 (66.0 to 87.5)	42.3 (30.6 to 54.6)	54.9 (44.2 to 65.4)	68.2† (52.4 to 81.4)	0.20 (0.05 to 0.35)	59.3 (50.5 to .67.6)
All household products stored at adult eye level or above [22]	90.0 (55.5 to 99.7)	88.5 (81.7 to 93.4)	37.5 (18.8 to 59.4)	99.1† (95.3 to 100)	0.48 (0.27 to 0.69)	88.6 (82.1 to 93.3)
All medicines and household products stored at adult eye level or above [18]	80.0 (28.4 to 99.5)	87.1 (80.3 to 92.1)	18.2 (5.2 to 40.3)	99.2† (95.5 to 100)	0.25 (0.04 to 0.47)	86.8 (80.2 to 91.9)
All medicines have child resistant caps or blister packs [1]	93.3 (86.7 to 97.3)	17.9 (8.9 to 30.4)	68.1‡ (59.8 to 75.6)	58.8 (32.9 to 81.6)	0.13 (0.00 to 0.27)	67.1 (59.2 to 74.3)
Any medicines been put in a container different from the one they came in [1]	33.3 (7.5 to 70.1)	98.0 (94.3 to 99.6)	50.0 (11.8 to 88.2)	96.1† (91.8 to 98.6)	0.37 (0.05 to 0.69)	94.4 (89.7 to 97.4)
All medicines kept in a locked medicine box [1]	50.0 (6.8 to 93.2)	82.8 (76.0 to 88.4)	6.9 (0.8 to 22.8)	98.5† (94.6 to 99.8)	0.08 (−0.06 to 0.22)	82.0 (75.2 to 87.6)
Medicines kept in fridge [1]	61.1 (43.5 to 76.9)	91.2 (84.8 to 95.5)	66.7 (48.2 to 82.0)	89.1† (82.3 to 93.9)	0.54 (0.38 to 0.70)	84.5 (77.9 to 89.7)
All household products have child-resistant caps [1]	71.9 (58.5 to 83.0)	35.6 (26.4 to 45.6)	38.0 (28.8 to 47.8)	69.8† (55.7 to 81.7)	0.06 (−0.06 to 0.19)	48.5 (40.5 to 56.4)
Any household products put in container different from the one they came in [0]	6.3 (0.2 to 30.2)	97.9 (94.1 to 99.6)	25.0 (0.6 to 80.6)	90.5† (84.8 to 94.6)	0.06 (−0.12 to 0.24)	88.9 (83.0 to 93.3)
Safety catch/lock on fridge§ [1]	100 (2.5 to 100)	67.7 (48.6 to 83.3)	9.1 (0.2 to 41.3)	100† (83.9 to 100)	0.12 (−0.10 to 0.33)	68.8 (50.0 to 83.9)

[ ] missing values.

*Considered safe if stored at adult eye level (or above) or in drawers and cupboards with catches or locks, or if none stored in the house.

§Question only asked of people reporting storing medicines in fridge (n=33).

†A higher proportion of respondents over-reported than under-reported safe practice (NPV exceeds PPV).

‡A higher proportion of respondents under-reported than over-reported safe practice (PPV exceeds NPV).

In table 4, the κ coefficients indicated that two of the three safety practices relating to scalds had moderate agreement (kettle kept at back of kitchen surface, and safety gate across kitchen doorway) and one only had slight agreement (has cordless kettle or curly flex, κ=0.13). Sensitivity was high (≥70%) for all the three practices and specificity was high (≥70%) for two of the three practices.

**Table 4:** Sensitivity, specificity, predictive values, κ value and percentage agreement between the questionnaire responses and observations related to scalds

Practice	Sensitivity (95% CI)	Specificity (95% CI)	PPV (95% CI)	NPV (95% CI)	κ Value (95% CI)	% Agreement
Has cordless kettle or curly flex [2]	82.1 (75.1 to 87.7)	75.0 (19.4 to 99.4)	99.2‡ (95.8 to 100)	9.7 (2.0 to 25.8)	0.13 (−0.02 to 0.28)	81.9 (75.0 to 87.5)
Kettle kept at back of kitchen surface [1]	94.2 (88.4 to 97.6)	42.5 (27.0 to 59.1)	83.2‡ (75.9 to 89.0)	70.8 (48.9 to 87.4)	0.42 (0.26 to 0.59)	81.4 (74.5 to 87.1)
Safety gate across kitchen doorway [0]	79.4 (62.1 to 91.3)	85.2 (77.8 to 90.8)	58.7 (43.2 to 73.0)	94.0† (88.0 to 97.5)	0.57 (0.43 to 0.72)	84.0 (77.4 to 89.2)

[ ] missing values.

†A higher proportion of respondents over-reported than under-reported safe practice (NPV exceeds PPV).

‡A higher proportion of respondents under-reported than over-reported safe practice (PPV exceeds NPV).

Further analysis was undertaken to determine whether recent changes in practice could account for the discrepancies between reported and observed exposures. Findings using the adjusted figures were broadly similar to those from the main analysis.

Out of the 30 safety practices for which predictive values could be calculated, for 24 practices a higher proportion of respondents over-reported than under-reported safe practice (NPV exceeds PPV). For six practices a higher proportion of respondents under-reported than over-reported safe practice (PPV exceeds NPV).

## Discussion

This research provides evidence about the validity of self-reported practices from a home safety questionnaire and indicates which questions could be reliably used in future research and practice. It is the largest study of its kind, and shows that for this questionnaire, the sensitivity, specificity and positive and negative predictive value of self-reported practices vary between safety practices. Eighteen of the 30 practices had at least a fair degree of chance-corrected agreement, but only three practices had substantial agreement (has a stair gate at the top of stairs, has stair gate at the bottom of stairs, and stairs are carpeted). Poison prevention practices appeared to have poorer agreement than falls or scald prevention practices. Overall, more respondents over-reported safe practice than under-reported them, but despite this, two-thirds of the questions had a high specificity. These questions will be useful for practitioners for identifying households with unsafe practices who may benefit from home safety interventions and for researchers who wish to use measures of unsafe practice as exposures or outcomes.

Sensitivity was high (>70%) for 19 out of 30 safety practices. Questions with high sensitivity will be useful for practitioners to identify families who do not need home safety interventions because they already have safe practices, and for researchers wishing to use measures of safe practice as exposures or outcomes. Questions with high sensitivity and specificity, of which there were 10 in our study, will result in least misclassification of exposures in observational studies.^25^ Only seven out of 30 of the observed practices had a high PPV (>70%). It is of note that six of these related to a standard item of equipment, for example, a cordless kettle or safety gate. Previous studies have also found responses about safety devices, and were reported more accurately than those about practices not requiring devices.^14^ ^17^ One study also reported that some parents may have ‘experienced some confusion’ over certain safety devices.^14^ This was noted by our researchers in relation to some items, for example, some families were unsure what furniture corner covers and stationary play centres were. Future questions on these will need more description, and possibly pictures, to reduce such confusion.

Previous studies have suggested potential reasons for disagreements between self-reports and observations, some of which may be applicable to our study.^11^ ^16^ ^20^ ^22^ These include providing socially desirable responses such as, for safe storage of medicines, and having inaccurate perceptions about safety. Disagreements could also be due to changes to safety practices or movement of hazards or safety equipment such as baby walkers or stair gates, between questionnaire completion and observation. Our analysis allowing for such changes did not substantially alter our results, suggesting this is unlikely to explain our findings.

Previous validation studies have reported varied findings.^14^ ^16^ ^17^ ^20^ ^22^ There are substantial methodological differences between these studies and our current study which may explain some of the variation. For example, one study compared only five practices, and the researchers read the questions to the families in their homes and then immediately observed the home,^17^ whereas, another undertook telephone interviews about four practices, and then researchers followed this up with observations 2–4 weeks later.^20^

The most comparable study to the research reported in this paper is our earlier study.^21^ That study reported greater consistency between self-reported data and observations (the PPV was 78% or above for 15 of the 16 practices). The two studies were similar in terms of using self-completion questionnaires covering a range of home safety topics; observations were undertaken at least a day after the self-reports; and researchers observing were blind to questionnaire responses. Although the questionnaire we used in the current study was based on the earlier one, it had notable differences in terms of size, overall design and the complexity of certain questions. For example, the later questionnaire assessed a much larger number of safety practices, safety equipment use and hazards (approximately 16 pages of questions compared with 7 pages), had more questions per page, had less white space, less vertical flow, and had more complex matrix questions.^6^ ^26^ These differences may explain some of the variations in the findings from the two studies.

This study had a number of strengths including being a large multisite study, with researchers at each site who were trained for the home observations, and blind to questionnaire responses at the time of the observations. Parents were not informed that the purpose of the visit was to validate a questionnaire, but they may have remembered their responses to the questionnaire, and this may have influenced their behaviour before or on the day of the observation.

Our comparison of characteristics of participants and non-participants suggests some differences between these two groups. Although we found no significant difference for most characteristics, respondents with children aged less than 1 year were less likely, and those with children aged 13–36 months were more likely to participate in home observations. Respondents with male children, those in single parent households and in households with a higher number of adults out of work were more likely to take part in home observations. This suggests our findings are likely to be generalisable to higher-risk households which is important, as these are likely to be targeted for home safety interventions and are likely to be the population of interest for injury researchers. Practitioners and researchers wishing to use our questions in populations at low risk of injury should consider further validation of our questions.

κ Statistics were used in this study as they can provide a general measure of agreement corrected for agreement due to chance, but we were aware that they are influenced by the prevalence of the exposure and are susceptible to bias.^27^ ^28^ Additionally, we calculated a range of validation measures comparing reported safety practices with observed values, so that these can be used to inform a range of further studies. A final potential limitation of the validation of the questionnaire is that it was not possible to undertake observations immediately after questionnaire completion. However, we endeavoured to keep the time period between the respondents completing the questionnaire and the home visits as short as was practical. Additionally, our analyses suggest changes made between questionnaire completion and home visit do not explain disagreements between observed and self-reported safety practices.

The motivation for this study was to validate exposures used in a series of case-control studies. Our findings suggest there will be less misclassification of exposures for falls prevention and scalds prevention practices than for poison prevention practices. Where exposures are misclassified, and where this does not occur differentially between cases and controls, measures of association between exposures and injury will be biased towards unity.^25^ This would mean that any significant associations we found were conservative estimates of the ‘true’ association. An assessment of the extent to which under-reporting and over-reporting of safety practices varies between cases and controls will be reported elsewhere.

For some safety practices, further research is needed to develop better measures, for example, in relation to poison prevention practices, particularly those investigating safe storage. The use of more individual questions rather than matrix questions should be examined, as this format may make the questionnaire easier for respondents to understand and complete.

In conclusion, future home safety researchers and practitioners using self-completed questionnaires or home safety checklists may wish to use some of the questions that were part of our research tool. In choosing questions, they should take heed of our results and be cognisant of the known principles of questionnaire design.^6^ ^7^ ^9^ ^26^ Questions with a high specificity will be useful for practitioners who want to identify families who do not have certain safe practices and who would benefit from home safety interventions, and for researchers wishing to use them as measures of exposure or outcome.

**What is already known on this subject**

- The validity of self-reported data is an important issue in injury research.
- Different types of research tools have been validated in different settings.
- Few studies have attempted to validate postal questionnaires.

**What this study adds**

- This study found that the validity of self-reports from a questionnaire varied with safety practice and indicates which questions could be reliably used in future research and practice.
- Our questions with a high specificity will be useful for practitioners who want to target families with home safety interventions.
